# Electronic Alert System Significantly Increases HBV Screening Rates Before Immunosuppressive Treatments

**DOI:** 10.5152/tjg.2023.22297

**Published:** 2023-05-01

**Authors:** Aydın Şeref Köksal, Bilal Toka, Mustafa Sadeçolak, Şencan Acar, Ahmet Tarık Eminler, Mukaddes Tozlu, Oğuz Karabay

**Affiliations:** 1 Department of Gastroenterology, Sakarya University Faculty of Medicine, Sakarya, Turkey; 2 Department of Gastroenterology, Health Sciences University Konya Training and Research Hospital, Konya, Turkey

**Keywords:** Alert program, chemotherapy, HBV, hematology, immunosuppressive treatment, oncology

## Abstract

**Background:**

Hepatitis B Virus (HBV) screening rates before starting immunosuppressive treatments are suboptimal. The aim of the study was to evaluate the efficacy of a new electronic alert system in increasing HBV screening rates.

**Methods:**

The electronic alert system, HBVision2, identifies patients at risk of HBV reactivation when a pre-determined International Classification of Diseases (ICD)-10 code is entered into the hospital’s database or immunosuppressive treatment is prescribed. The system evaluates the prior Hepatitis B Surfage Antigen (HBsAg) and anti-Hepatitis B Core Immunglobulin G (HBc IgG) results and sends an alert code to the clinician for screening if serology is not completely available or consult a specialist in case of positive serology. The HBV screening and consultation rates of patients before (control group) and after HBVision2 were retrospectively compared. The clinical course of unscreened and/or unconsulted patients was determined, and the clinical efficacy of HBVision2 in preventing HBVr was predicted.

**Results:**

Control group included 815 patients (52.6% male, mean age: 60 ± 12, 82.5% with oncologic malignancy) and study group included 504 patients (56% male, mean age: 60 ± 13, 91.4% with oncologic malignancy). Groups were similar with respect to gender, mean age, and HBVr risk profile of the immunosuppressive treatment protocols. Overall, both HBsAg (from 55.1% to 93.1%) and anti-HBc IgG screening rates significantly increased (from 4.3% to 79.4%) after the electronic alert system (*P*  < .001, for both). Consultation rates of anti-HBc IgG-positive patients significantly increased from 40% to 72.7% (*P*  = .012). HBVr developed in 2 patients (2.6%) who were not screened and/or consulted after the alert system. Alert program prevented the development of HBVr in 10 patients (1.9%) of the study group and decreased the development of HBVr by 80%.

**Conclusion:**

Electronic alert system significantly improved HBsAg and anti-HBc IgG screening rates before starting immunosuppressive treatment and prevented the development of HBVr to a great extent. However, screening rates are still below optimal and need to be improved.

**Main Points**

- HBV screening rates before starting immunosuppressive treatments (IST) are suboptimal.
- A new electronic alert system (HBVision2) which alerts the primary clinicians to screen for HBV before starting IST and consult an HBV specialist in patients with positive HBV serology has been developed.
- New electronic alert system significantly improved HBsAg and anti-HBc IgG screening rates before starting immunosuppressive treatments and prevented the development of HBVr to a great extent (80%).

## Introduction

Patients with current or prior exposure to Hepatitis B Virus (HBV) are at risk for HBV reactivation (HBVr) when they receive immunosuppressive treatments (IST). The consequences of HBVr can range from subclinical increase in HBV DNA levels to hepatitis, liver failure, and death.^1^ HBV reactivation can also lead to early cessation of IST and thus delay the treatment of the underlying disease.

Prophylactic antiviral therapy is highly effective in reducing the risk of HBVr due to IST in Hepatitis B Surfage Antigen (HBsAg)- and/or anti-Hepatitis B Core Immunglobulin G (HBc IgG)-positive patients.^2-4^ Current guidelines recommend HBV screening prior to IST treatment in all patients,^5-7^ those at high risk for HBV infection^8-10^ or at moderate to high risk for HBVr,^9,10^ and antiviral prophylaxis is recommended to patients at moderate or high risk for HBVr.^5,6,9,10^ However, HBV screening rates are far beyond optimal according to the real-world data. For example, a study from a major US hospital reported that among 11 959 cancer patients receiving chemotherapy between 2012 and 2015, only 15.5% were screened for both HBsAg and anti-HBc IgG.^11^ Lack of awareness is probably the most important reason for these suboptimal HBV screening rates.^12^

We developed a new electronic alert system (HBVision2) which alerts the primary clinicians to screen for HBV before starting IST and consult to an HBV specialist in patients with positive HBV serology. The aim of the study was to evaluate the efficacy of this new alert system in increasing the HBV screening rates before starting IST.

## Materials and Methods

### Computer Alert System

**Figure 1.**
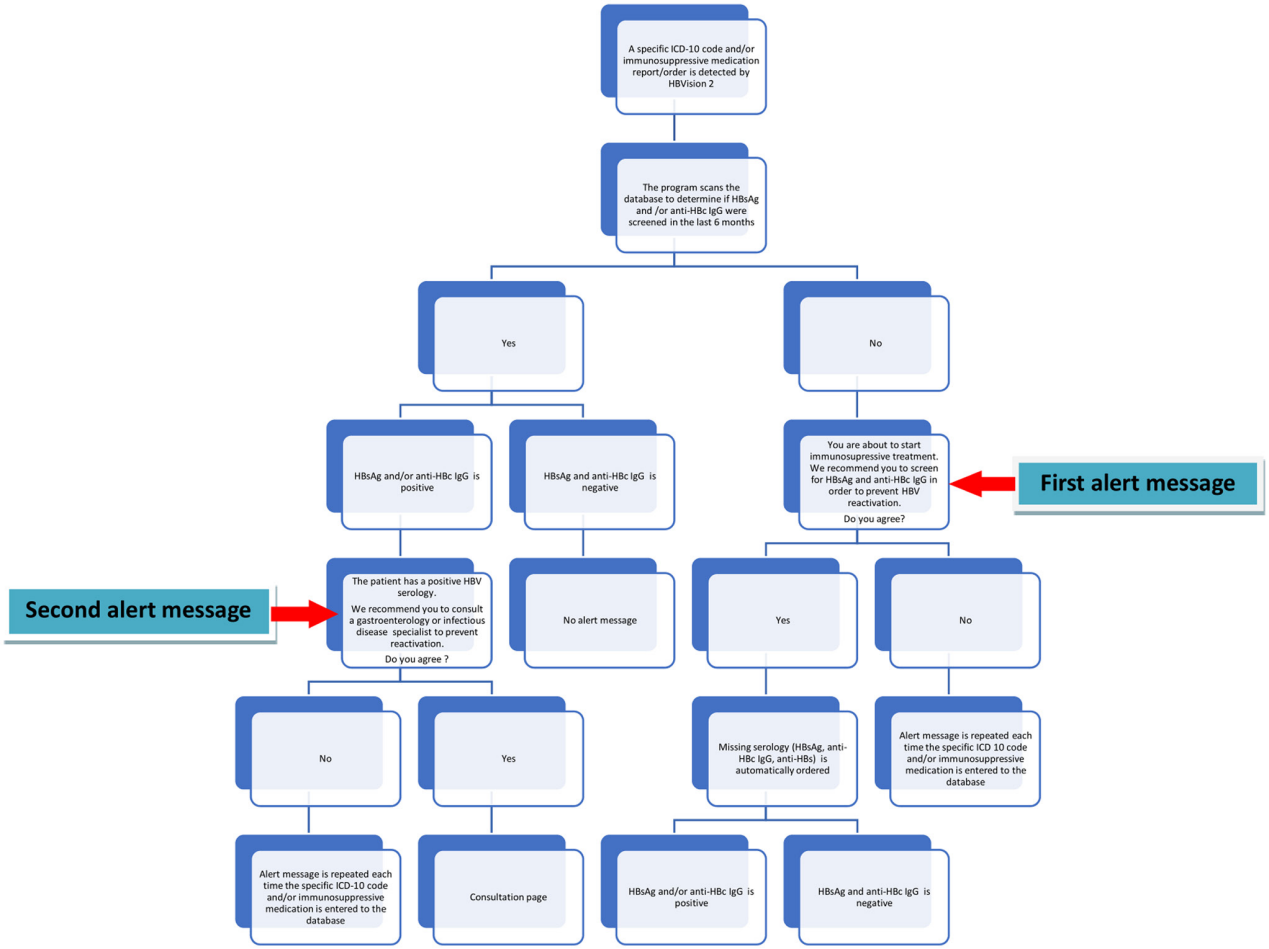
HBV screening algorithm of the electronic alert system (HBVision2).

The new electronic alert system ‘’HBVision2’’ is a modification of HBVision1 which was uploaded to our hospital’s database in 2016.^13^ The new system is more user-friendly than the previous one and contains shortcuts for ordering the missing HBV serologic markers and requesting consultation from an HBV specialist in case of positive HBV serology. HBVision2 was uploaded to our hospital’s database in 2019 by the hospital’s IT staff, at no cost. Briefly, the system identifies patients at risk of HBVr in 2 different ways. First, it identifies pre-determined International Classification of Diseases (ICD)-10 codes of diseases (oncologic, hematologic, and others) which may require IST. Second, it identifies IST prescriptions. The specific ICD-10 codes and immunosuppressive medications were initially determined at a consensus meeting attended by oncology, hematology, gastroenterology, and infectious disease specialists and later on, periodically updated by the infectious disease specialist of our hospital. When the system identifies a patient at risk of HBVr according to the presence of the above-mentioned criteria, it analyzes the HBV serology (HBsAg and anti-HBc IgG) of the patient within the last 6 months. If any of the HBV serologic markers (HBsAg and anti-Hbc IgG) are missing, the computer sends the following alert code to the clinician: ‘’You are about to start IST. We suggest you to screen for HBsAg, anti-HBc IgG and anti-HBs to prevent HBVr. Do you agree?’’ If the clinician hits the “Yes” button, the system automatically sends the laboratory test order for the missing HBV marker(s). If the patient has positive HBsAg and/or anti-HBc IgG at baseline or after screening with the computer alert system, the system sends the following alert code to the clinician: ‘’The patient has positive (+) HBV serology. Please consult to a gastroenterology or infectious disease specialist to assess for HBVr risk. Do you want to consult now?’’ (Figure 1). When the patient consults with an HBV specialist, HBVr risk is evaluated and the alert system is terminated by the consultant if a decision to start prophylaxis or follow-up is given. However, in most of the isolated anti-HBc-positive cases, decision is given according to the HBV DNA results. In this case, the consultant does not terminate the system and the system continues to send alert codes to the primary clinician at each visit until HBV DNA levels are evaluated, and a final decision is made by the consultant. If the clinician does not screen for HBV or consult the patient with positive serology to an HBV specialist, the system sends an alert message to the primary clinician each time the ICD-10 code is entered into the database or IST is prescribed.

### Study Population and Parameters

The study group included oncology and hematology patients who received IST between August 2020 and February 2021. Control group included oncology and hematology patients who received IST within 1 year before the first electronic alert system (HBVision1) was uploaded to the hospital’s database. Patients <18 years, those with a prior diagnosis of chronic HBV infection, and those who were lost to follow-up were excluded from the study. Demographic variables, underlying primary diseases, IST protocols, and HBVr risk profiles of the study and control groups were determined by reviewing the hospital’s database. HBV screening rates and consultation rates by a HBV specialist were compared between the study and control groups. These parameters were also compared in subgroups which were categorized according to the underlying disease (oncologic vs. hematologic) and HBVr risk profile of the IST protocol. Finally, the clinical course of unscreened patients and patients with positive serology but not consulted an HBV specialist were reviewed for the development of HBVr and its clinical consequences. All of the oncology and hematology specialists were unaware of the collection of the data and they were blind to the study. The study protocol conforms to the ethical guidelines of the Declaration of Helsinki and it was approved by the institutional review board of Sakarya University, Faculty of Medicine (E-715 22473 -050. 01.04 -1481 3/93).

### Definitions

HBVr was defined as a ≥1log increase in HBV DNA levels from baseline values or reappearance of HBV DNA in a patient with an undetected baseline HBV DNA. HBV flare was defined as HBVr associated with at least a 3-fold increase in serum ALT levels above the patient’s baseline values or upper limit of the reference range, with or without signs and symptoms of hepatitis. HBVr risk profiles of the treatment protocols (high, moderate, or low risk) were categorized according to the American Gastroenterological Association (AGA) guideline.^9^ Interruption of IST was defined as delaying the IST by more than 7 days. Discontinuation of IST was defined as failure to restart IST after interruption.

### Outcomes of the Study

The primary outcome of the study was to evaluate the efficacy of HBVision2 in increasing HBV screening rates before starting IST. The secondary outcomes were to evaluate the consultation rates by an HBV specialists in case of positive serology, the efficacy of alert system in preventing the development of HBVr, and the clinical course of unscreened or unconsulted patients.

### Serologic Tests

HBsAg, anti-HBc, HBeAg, anti-HBe, and anti-HBs were tested by commercial assays (Vitros, Upjohn, Germany). HBV-DNA was quantified by AmpliPrep/COBAS® TaqMan® (Roche Diagnostics GMBH, Mannheim; Germany, range: 15-100 000 000 IU/mL).

### Statistical Analysis

Statistical analyses were performed using Statistical Package for the Social Sciences 22.0 (IBM Corp.; Armonk, NY, USA). Descriptive statistics (mean with standard deviation and simple proportions) were used to present patient characteristics and serological results. Continuous variables were compared with Student’s *t* test. Categorical variables were compared with chi-square test. Statistical significance was set at *P* <.05.

## Results

### Clinical Characteristics of the Study and Control Groups

In total, 508 patients received IST within 6 months after the electronic alert system was uploaded to the hospitals’ software. Four of them were excluded from the study because of a prior diagnosis of chronic HBV infection or hepatitis. The remaining 504 patients constituted the study group; 56% of the study group were male, with a mean age of 60 ± 13 years. The underlying diseases were oncologic malignancy in 461 patients (91.4%) and hematologic malignancy or diseases in 43 patients. Eight-hundred and twenty-nine patients received IST within 1 year before the alert system. Fourteen of them were excluded from the study because of a prior diagnosis of chronic HBV infection or hepatitis. The remaining 815 patients (52.6% male, mean age: 60 ± 12) constituted the control group. The underlying diseases were oncologic malignancy in 673 patients (82.5%) and hematologic malignancy or diseases in 142 patients. HBVr risk profile of the IST protocols was high risk in 21 and 54 patients and moderate risk in 483 and 761 patients of the study and control groups, respectively. There was no significant difference between the gender, mean age, and HBVr risk profile of the IST protocols of the study and control groups (Table 1).

### HBV Screening Rates

**Figure 2.**
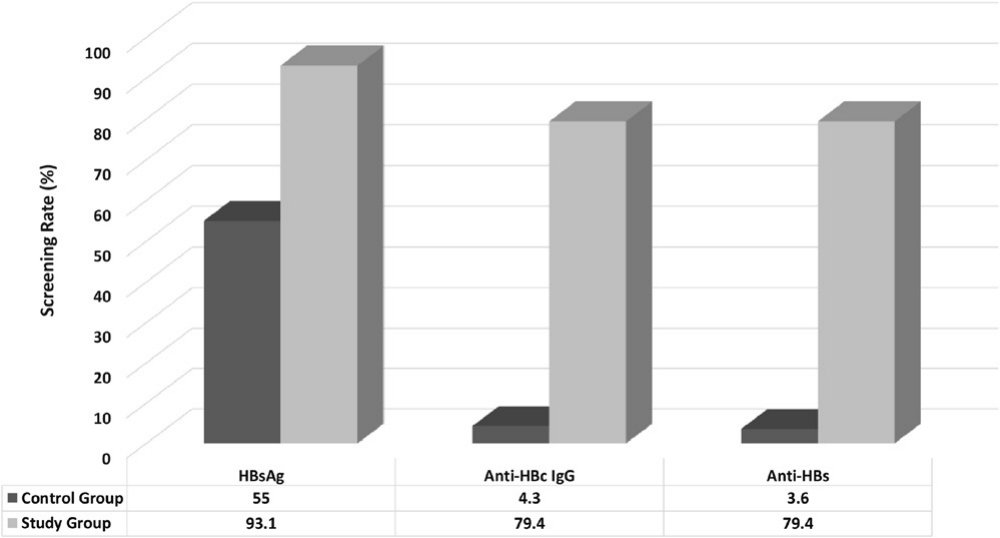
HBV screening rates before and after HBVision2.

In the control group (n = 815), 449 patients (55.1%) were screened only for HBsAg and 35 patients (4.3%) were screened for both HBsAg and anti-HBc IgG. Anti-HBs was screened in 16 (2%) patients. After the alert system, 469 patients (93.1%) were screened for HBsAg and 400 patients (79.4%) were screened for HBsAg, anti-HBc IgG, and anti-HBs. There was a significant increase in the HBsAg (55.1% vs. 93.1%), anti-HBc IgG (4.3% vs. 79.4%), and anti-HBs screening rates (2% vs. 79.4%) after the alert system (*P*  < .001, for all) (Figure 2).

### HBV Screening Rates According to the Underlying Diseases and HBVr Risk Profiles

In the oncology patients (n = 673), 317 (47.1%) were screened only for HBsAg, 7 patients (1%) were screened for both HBsAg and anti-HBc IgG, and 4 patients (0.4%) were screened for anti-HBs before the alert system. After the alert system (n = 461), 429 patients (93.1%) were screened for HBsAg, 366 patients (79.4%) were screened for HBsAg, anti-HBc IgG, and anti-HBs. There was a significant increase in the screening rates of HBsAg (from 47.1% to 93.1%), anti-HBc IgG (from 1% to 79.4%), and anti-HBs (from 0.4% to 79.4%) after the electronic alert system (*P*  < .001, for all).

**Figure 3.**
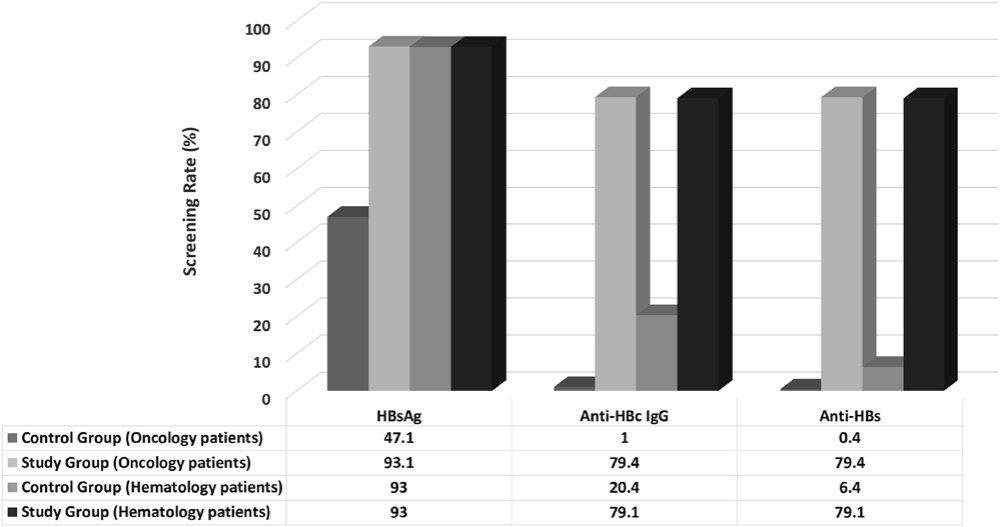
HBV screening rates of the oncology and hematology patients in the control and study groups.

In the hematology patients (n = 185), 132 patients (93%) were screened only for HBsAg, 29 patients (20.4%) were screened for HBsAg and anti-HBc IgG, and 12 (6.4%) were screened for anti-HBs before the alert system. After the alert system (n = 43), 40 patients (93%) were screened for HBsAg and 34 patients (79.1%) were screened for HBsAg, anti-HBc Gig, and anti-HBs. There was a significant increase in the screening rates of anti-HBc IgG (from 20.4% to 79.1%) and anti-HBs (from 6.4% to 79.1%) after the alert system (*P*  < .001)(Figure 3).

**Figure 4.**
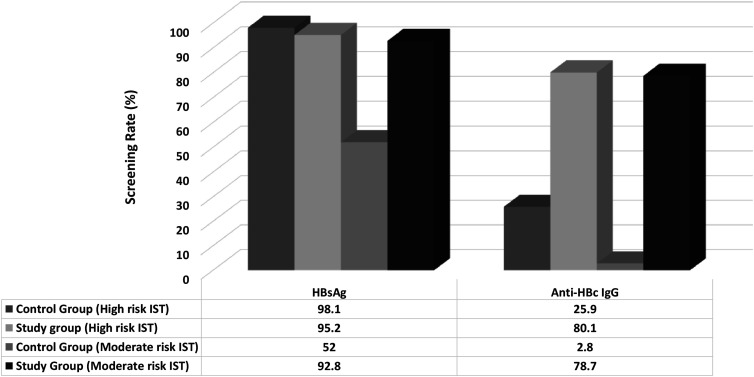
HBV screening rates according to the HBVr risk profile of the treatment protocols in the control and study groups.

When the screening rates were analyzed according to the HBVr risk profile of the IST protocols, in patients receiving a high risk IST protocol, there was no significant difference between the HBsAg screening rates of patients before (98.1%) and after the alert system (95.2%), but anti-HBc IgG screening rates significantly increased from 25.9% to 80.1% (*P*  < .001). However, in patients receiving a moderate risk treatment protocol, both HBsAg (from 52% to 92.8%) and anti-HBc IgG (from 2.8% to 78.7%) screening rates significantly increased after the alert system (*P*  < .001, for both) (Figure 4).

### Consultation Rates in Patients with a Positive Serology

In the control group, 13 patients were HBsAg-positive and 15 were anti-HBc IgG-positive. Nine (69.2%) HBsAg-positive and 6 (40%) anti-HBc IgG-positive patients consulted an HBV specialist. In the study group, 10 patients were HBsAg-positive and 154 were anti-HBc IgG-positive. Nine (90%) HBsAg-positive and 112 (72.7%) anti-HBc IgG-positive patients consulted an HBV specialist. Although there was no significant difference between the consultation rates of the HBsAg-positive patients before and after the alert system (*P*  = .25), consultation rates of the anti-HBc IgG positive patients significantly increased after the alert system (*P*  = .012).

### Clinical Course of the Patients with a Positive Serology

Out of 10 HBsAg-positive patients, 9 (90%) consulted an HBV specialist. HBVr risk profile of the treatment protocols was high risk in 2 patients and moderate risk in 7 patients. One patient was HBV DNA-positive (5650 IU/mL). All 9 patients received antiviral prophylaxis with tenofovir disoproxil fumarate (n = 7) or entecavir (n = 2). One patient died due to disease progression. None of the 9 patients developed HBVr during a mean follow-up of 9 months (range: 7-13). One HBsAg-positive patient with colon carcinoma did not consult an HBV specialist and received a moderate risk IST. She did not develop either asymptomatic increase in serum transaminase levels or HBVr during a treatment period of 11 months.

Out of 154 isolated anti-HBc IgG-positive patients, 112 (72.7%) consulted an HBV specialist. HBVr risk profile of the treatment protocols was high risk in 9 patients and moderate risk in 103 patients. All of them were HBV DNA-negative and received antiviral prophylaxis with either tenofovir disoproxil fumarate (n = 87), entecavir (n = 23), or tenofovir alafenamide (n = 2). None of them developed HBVr during a mean follow-up period of 10.1 months (range: 7-13). Forty-two isolated anti-HBc IgG-positive patients did not consult an HBV specialist. HBVr risk profile of the treatment protocols was high risk in 2 and moderate risk in 40 patients. One of them developed severe acute hepatitis after receiving R-CHOP for lymphoma. Teneofovir disoproksil fumarate (TDF) was started and chemotherapy was interrupted for 3 months. Most of the remaining patients (n = 38) were followed by only liver enzymes and none of them developed an increase in liver tests that were related to HBVr.

### Clinical Course of the Unscreened Patients

In the study group, 35 patients were not screened for both HBsAg and anti-HBc IgG. One of them developed HBV flare after receiving cisplatin and etoposide for lung cancer. TDF was started and chemotherapy was not interrupted. One hundred four patients were not screened for anti-HBc IgG. They were followed by liver enzymes and none of them developed an increase in liver tests that were related to HBVr during a mean follow-up period of 10.2 (range 7-13) months.

#### Efficacy of Alert Program in Preventing the Development of HBVr

HBVr developed in 2 patients (2/78 : 2.6%) who were not screened and/or consulted after the alert system. The rate of unscreened and/or unconsulted patients in the control group was 94.1%. Assuming that this rate would be the same in both the control and study groups, had there been no alert program, we predict that HBVr would develop in an expected number of 12 patients (504 × 94.1% × 2.6%= 12) in the study group. Therefore, we conclude that our alert program prevented the development of HBVr in additional 10 patients (1.9%) of the study group and decreased the development of HBVr by 80%.

## Discussion

Our new electronic alert system significantly increased HBsAg (from 55.1% to 93.1%) and anti-HBc IgG (from 4.3% to 79.4%) screening rates before starting immunosuppressive treatments and decreased the development of HBVr after IST by 80%. However, we think that the screening rates still need to be improved.

HBVr after IST can cause severe hepatitis, liver failure, and death in more than 10% of the patients.^14,15^ Therefore, HBV screening before starting IST is recommended to determine the HBVr risk profile of the patient and to determine whether he/she needs antiviral prophylaxis. Despite the strong recommendations of major international organizations,^5-10^ HBV screening rates are still far below optimal according to real-world data. For example, a study from the US reported that among 8005 patients undergoing cytotoxic chemotherapy between 2006 and 2011, only 16% were screened for HBV infection. Screening rates were even lower (10.6%) in oncology patients.^16^ An international survey of the membership of the American Association for the Study of Liver Diseases reported that among 188 patients with HBVr, only 39.9% were screened for both HBsAg and anti-Hubs.^15^ In the current study, HBV screening rates were also very low and only 4.3% of the patients were screened for both HBsAg and anti-HBc IgG before starting IST. On the other hand, the use of IST significantly increased in recent years parallel to increase in cancer incidence rates and the use of biological agents in various fields of medicine. Therefore, the problem needs an urgent solution.

Lack of awareness, heavy workload of clinicians, financial burden, and fear of delay in starting chemotherapy are probably the most important reasons for these suboptimal HBV screening rates before starting IST. There are some ways to overcome these reasons. Multidisciplinary meetings can increase the awareness of the primary clinicians about HBVr and increase the communication between the primary and consultant clinicians. Electronic alert systems also increase the awareness of the clinicians and remind HBV screening, which is important, especially in busy clinics.

Up to our knowledge, there are only a few studies about the use of electronic alert systems for increasing HBV screening rates before starting IST.^13,17-20^ In a study from Spain, PRESCRIB project, an alert system increased HBsAg (from 47% to 94%) and anti-HBc IgG screening rates (from 29% to 85%) in patients receiving biologic agents.^17^ In a Japanese study, HBView, an alert system increased anti-HBc IgG screening rates from 71% to 84% in patients receiving chemotherapy.^18^ A recent study from Spain also reported significant increase in HBV and HCV screening rates from 60.5% to 87.8% in 420 hematology patients after electronic alert system.^20^ Our alert system also significantly increased HBsAg (from 55.1% to 93.1%) and anti-HBc IgG screening rates (from 4.3% to 79.4%) in patients receiving IST. However, our system had several important advantages over the previously defined ones. First, the previous alert systems identified patients at risk of HBVr only when an immunosuppressive medication was prescribed. However, our alert system has dual identification mechanism, and in addition to identifying the prescription of immunosuppressive medications, it also identifies pre-determined ICD-10 codes of oncologic and hematologic diseases. This dual identification leads to early identification of the patients at the time of diagnosis and hence saves time for the evaluation of HBVr risk profiles. It also serves as a double-check mechanism to prevent overlooking patients at risk of HBVr, as new ISTs are added to clinical practice very frequently and they may be overlooked by the alert system, if the list of IST is not updated frequently. Second, our alert system is much more user-friendly than the previous ones. The previous alert systems send alert messages to clinicians to search for the previous HBV serologic markers of the patients. Afterwards, the clinicians either code the serologic markers to the alert system for getting a suggestion about HBVr^17,20^ or follows the pre-determined suggestions on the alert page categorized according to different HBV serologies.^18^ In most of the previous alert systems, the clinicians had to order the missing serologic markers manually and follow the results themselves. In addition, there were no reminder messages. However, in the current alert system, the system automatically analyzes the previous HBV serology of the patients within the last 6 months and sends alert codes to the clinicians only if they are missing or positive. We think that this selectivity is important to prevent the development of alert fatigue in clinicians. In addition, if the clinician agrees to screen for HBV, the system automatically orders only the missing serology of the patients. The last but not the least, the current alert system sends reminder messages until HBV screening and consultation of the patients with positive serology are finalized.

Although all of the guidelines suggest antiviral prophylaxis in HBsAg-positive patients before starting moderate to high-risk IST, the management of isolated anti-HBc IgG-positive patients is controversial. Other than AGA,^9^ most of the guidelines suggest to start antiviral prophylaxis only in patients receiving B cell-depleting agents or undergoing stem cell transplantation.^5,8,10^ However, all of the isolated anti-HBc IgG patients should be checked for HBV DNA, and HBV DNA-positive patients should be managed similar to HBsAg-positive ones. HBV DNA-negative patients who will receive moderate to low risk treatments should be followed at 1-3 month intervals for HBVr. Despite these recommendations by major international guidelines, anti-HBc screening rates are significantly lower than HBsAg screening rates.^21^ In the current study, the alert system significantly increased the anti-HBc IgG screening rates from 4.3% to 79.4% and 72.7% of the cases with positive serology consulted by an HBV specialist. However, these figures were still below than those for HBsAg (93% for screening and 90% for consultation to an HBV specialist in case of positive serology). We think that there are several reasons for these low HBV screening rates despite the new electronic alert system. Lack of awareness, heavy workload of clinicians, and fear of delay in chemotherapy due to consultation are among the most important ones. We think that periodic multidisciplinary meetings, highlighting the importance of prophylaxis and follow-up in these patients, may help to increase these numbers to optimal levels.

HBV screening rates in solid organ tumors are usually significantly less than hematologic malignancies,^22^ as in the current study (1% vs. 20.4%). This difference may be due to the more frequent use of high-risk treatment protocols in hematology patients. The electronic alert system significantly increased HBV screening rates in oncology patients and there was no significant difference between the HBV screening rates of oncology (79.4%) and hematology (79.1%) patients.

Our study had several limitations. The study was a single-center study and only included relatively small number of oncology and hematology patients. The high HBV screening rates may be due to increased awareness of the clinicians due to first version of the computer alert system, which was uploaded to the hospital’s software in 2016. Moreover, the follow-up period was relatively short to make a conclusion about the clinical course of patients with positive serology. However, the primary aim of the study was to determine the efficacy of alert system in increasing the HBV screening rates which were significantly higher than those achieved after the first version of the alert system (68.6% for only HBsAg and 13.1% for both HBsAg and anti-HBc IgG).^13^

In conclusion, electronic alert system significantly improved HBsAg and anti-HBc IgG screening rates before starting immunosuppressive treatments and prevented the development of HBVr to a great extent (80%). However, screening rates are still below optimal and should be reinforced by additional efforts.

## Figures and Tables

**Table 1.** Clinical Characteristics of the Study and Control Groups

	Control Group (n = 815)	Study Group (n = 504)	*P*
Gender (M/F)	429/386	283/221	.23
Age (mean ± SD)	60.6 ± 12.8	60.4 ± 13	.7
Underlying diseases			
Oncologic malignancy (total)	673	461	
Lung cancer	177	120	
Breast cancer	128	93	
Colorectal cancer	101	83	
Others	267	165	
Hematologic malignancy/diseases (total)	142	43	
Lymphoma	62	19	
Multiple myeloma	30	11	
Myelodysplastic syndrome	4	5	
Acute myeloid leukemia	12	2	
Others	34	6	
HBVr risk profile of the treatment protocols, overall (high/moderate)	54/761	21/483	.07
Oncologic malignancy	8/665	2/459	.21
Hematologic malignancy/diseases (total)	46/96	19/24	.2

HBVr, HBV reactivation
